# Use of Dried Plasma Spots for HIV-1 Viral Load Determination and Drug Resistance Genotyping in Mexican Patients

**DOI:** 10.1155/2015/240407

**Published:** 2015-12-08

**Authors:** Juan Pablo Rodriguez-Auad, Othon Rojas-Montes, Angelica Maldonado-Rodriguez, Ma. Teresa Alvarez-Muñoz, Onofre Muñoz, Rocio Torres-Ibarra, Guillermo Vazquez-Rosales, Rosalia Lira

**Affiliations:** ^1^Unidad de Investigación Médica en Enfermedades Infecciosas y Parasitarias, UMAE Hospital de Pediatría, CMN Siglo XXI, Instituto Mexicano del Seguro Social (IMSS), Avenida Cuauhtémoc 330, Colonia Doctores, 06720 Mexico City, DF, Mexico; ^2^Department of Pediatric Infectious Diseases, Hospital Infantil de México Federico Gómez, Dr. Marquez 162, Colonia Doctores, 06720 Mexico City, DF, Mexico; ^3^Hospital de Infectología, UMAE Centro Medico Nacional “La Raza”, Instituto Mexicano del Seguro Social (IMSS), Avenida Jacarandas Esquina Vallejo S/N, Colonia La Raza, 02990 Mexico City, DF, Mexico

## Abstract

Monitoring antiretroviral therapy using measurements of viral load (VL) and the genotyping of resistance mutations is not routinely performed in low- to middle-income countries because of the high costs of the commercial assays that are used. The analysis of dried plasma spot (DPS) samples on filter paper may represent an alternative for resource-limited settings. Therefore, we evaluated the usefulness of analyzing DPS samples to determine VL and identify drug resistance mutations (DRM) in a group of HIV-1 patients. The VL was measured from 22 paired plasma and DPS samples. In these samples, the average VL was 4.7 log_10_ copies/mL in liquid plasma and 4.1 log_10_ copies/mL in DPS, with a correlation coefficient of *R* = 0.83. A 1.1 kb fragment of HIV* pol* could be amplified in 14/22 (63.6%) of the DPS samples and the same value was amplified in plasma samples. A collection of ten paired DPS and liquid plasma samples was evaluated for the presence of DRM; an excellent correlation was found in the identification of DRM between the paired samples. All HIV-1* pol* sequences that were obtained corresponded to HIV subtype B. The analysis of DPS samples offers an attractive alternative for monitoring ARV therapy in resource-limited settings.

## 1. Introduction

The prevalence of HIV-1 infection has been increasing all over the world, especially in low- and middle-income countries (LMICs). A 2013 report issued by the Joint United Nations Programme on HIV and Acquired Immune Deficiency Syndrome (UNAIDS) estimates that an average of 35.3 million people (range: 32.2 million–38.8 million) were living with HIV at the end of 2012 [1] and that 5.25 million people in LMICs were receiving antiretroviral (ARV) therapy [2]. Despite the accessibility of ARV therapy in these countries, the widespread monitoring of HIV treatment efficacy by evaluating viral load (VL) levels and analyzing of drug resistance (DR) has been suboptimal [3]. In contrast, in high-income countries (HICs) where the treatment of HIV is more closely monitored the emergence of HIV mutations that confer DR to ARV drugs has increased [4].

In HICs, the monitoring of ARV therapy using VL and genotypic resistance testing is essential to determining cases of treatment failure [5]; the quantification of VL serves as an indicator for whether ARV therapy leads to success or failure in HIV infected patients [6]. To limit the emergence of resistance to ARV drugs, patients undergoing treatment for HIV should ideally undergo periodic virological monitoring, such as VL and genotypic resistance testing, to identify cases in which therapy has failed and to avoid the accumulation of drug resistance mutations (DRM) [7].

The emergence of drug-resistant HIV remains a significant obstacle to the long-term success of therapy; however, it is costly to monitor patients by screening for VL and ARV drug resistance. A portion of this cost stems from the logistics that are involved in sample collection and transport [8]. Because of both the expenses and the logistic challenges that arise during sample collection and transportation from points of care to reference laboratories, VL and genotypic resistance tests remain unavailable to the majority of HIV infected individuals in resource-limited settings [9].

The use of DPS provides an attractive alternative for monitoring ARV therapy in HIV patients in LMICs [11], as DPS samples can be shipped from distant point-of-care clinics to central laboratories [12], and not only the cost reduction is in the collection and transport process, but also in the sample processing using an in-house assay. Dried blood, plasma, and serum spots (DBS, DPS, and DSS, resp.) have been successfully used to quantify viral RNA and evaluate genotypic drug resistance [5, 7, 13, 14]; however, select limitations and challenges continue to inhibit their practical use. These limitations include their lower limits of detection, the instability of nucleic acids in long-term storage, and the interference of proviral DNA. There is also the potential to overestimate the VL measurement and amplification success of DBS samples [14]. Although the World Health Organization (WHO) accepts the use of DBS for VL monitoring, in the case of evaluating genotypic resistance mutations, there are still several technical and interpretation issues to resolve. In the majority of the research conducted to date, DBS samples have been used to measure VL and perform genotyping; however, the best available sample for these measurements is actually plasma. We have previously reported only a 38.6% amplification success rate using the ViroSeq genotyping assay on DBS samples, which was likely due to either the PCR inhibitors that are present in erythrocytes or to sample storage conditions [15]. In this study, we evaluated the usefulness of analyzing DPS samples to determine viral load (VL) and identify drug resistance mutations in Mexican patients with HIV-1 infection.

## 2. Materials and Methods

### 2.1. Study Population

The study protocol was approved by the Ethics Committee and the Institutional Review Boards of the IMSS. Written informed consent was obtained from each participant. A group of 22 adults who were already infected with HIV-1 and receiving ARV therapy were enrolled in our study over the course of February 2009 to March 2010. These patients were receiving healthcare at the La Raza Infectious Diseases Hospital, a tertiary referral center of the IMSS.

### 2.2. Preparation of Plasma Samples and DPS

An 8 mL blood sample was collected from each of the 22 patients who were infected with HIV. The blood was collected into EDTA-coated sampling tubes. These samples were then centrifuged to isolate the liquid plasma, which was subsequently stored at −70°C until testing. DPS specimens were made by spotting 50 *μ*L of plasma per circle on filter paper (Schleicher & Schuell 903; Keene, NH, USA), which were then dried for 2 h at RT. The DPS samples were individually placed into small plastic bags with a sachet of desiccant (WA Hammond Drierite Co., Ltd., USA) and were stored at −20°C for an average of one week. One sample (HIV-22) was stored for 910 days at −20°C.

### 2.3. Viral Load Measurement

The RNA-HIV-1 VL in plasma and DPS were determined using the Amplicor HIV-1 monitor test, version 1.5 (50 to 750,000 copies/mL; Roche Diagnostics, Indianapolis, IN), according to the manufacturer's instructions. To measure RNA-HIV VL in DPS samples, a modification in the nucleic acid extraction was used as previously reported [15]. Briefly, two spots per sample were eluted in 100 *μ*L of elution buffer. Because the initial RNA volumes of plasma and DPS were different (500 *μ*L and 100 *μ*L, resp.), we multiplied the VL value obtained from DPS by 5 to normalize the data.

### 2.4. Nucleic Acid Extraction

RNA was extracted from the DPS samples using a NucliSens HIV-1 QT assay, NASBA Diagnostics procedure (bioMerieux, Inc., Durham, NC). Two whole spots, containing 100 *μ*L of dried plasma (50 *μ*L per spot), were cut with scissors and were transferred to a tube containing 9 mL of NucliSens lysis buffer (Organon Teknika, Durham, NC, USA), following incubation at RT under gentle rotation for 1 h. The nucleic acids were then extracted according to the manufacturer's instructions and resuspended in 100 *μ*L of elution buffer for storage at −70°C until use. The RNA from the plasma samples was extracted using the QIAamp UltraSens Virus Kit (Qiagen) according to the manufacturer's instructions.

### 2.5. PCR Amplification for Genotypic Drug Resistance Testing

We used the HIV-1 genotype assay that was previously reported by Inzaule et al. to validate the use of DPS samples in the genotype resistance assay [16]. A collection of primers that were previously described by Zhou et al. was used to amplify the section of the HIV-1* pol* gene that encoded amino acids 6 to 99 of the protease (PR) region and codons 1 to 251 of the reverse transcriptase (RT) region of the HIV-1 genome [9]. Briefly, 10 *μ*L samples of nucleic acid extracts isolated from either plasma or DPS were assessed by one-step RT-PCR using the PRTM-F1 outer primer pair, which is a 1 : 1 (wt/wt) combination of two forward primers, F1a (positions 2057 to 2085) and F1b (positions 2068 to 2092), RT-R1 (reverse, positions 3370 to 3348), and the SuperScript II one-step RT-PCR system with Platinum* Taq* high-fidelity polymerase, according to the manufacturer's protocol (Invitrogen, Carlsbad, CA). For nested PCR, 8 *μ*L of the RT-PCR product was used with the inner forward primer PRT-F2 (positions 2243 to 2266) and reverse RT-R2 (positions 3326 to 3304), 1x Phusion HF Buffer, 2 mM MgCl_2_, 0.4 mM dNTP, and 0.5 U of Phusion High-Fidelity DNA Polymerase (BioLabs) to amplify an amplicon of approximately 1.1 kb.

### 2.6. Sequencing Drug Resistance Mutations (DRM)

The 1.1 kb PCR products obtained from the RT-PCR were purified and directly sequenced using the BigDye Terminator Version 3.1 Cycle Sequencing Kit in an ABI 3100 sequencer (Applied Biosystems, Carlsbad, CA) using PRT-F2 and RT-R2 primers. Confirmation of base calling and sequence editing was conducted using CLC bio 6.0 version software. The presence of DRM in the PR and RT was identified using the Stanford genotyping drug resistance interpretation algorithm (v.4.2.6).

### 2.7. Phylogenetic Analysis

Phylogenetic analysis was performed using the neighbor-joining method and the reliability of the branching orders was assessed by using the bootstrap approach (1000 replicates) with Mega 6.06 software [10].

### 2.8. Statistical Analysis

The Pearson coefficient was used to determine the VL association between plasma and DPS samples. Student's *t*-test was used to compare VLs between paired samples. A *p* value of <0.05 was considered significant.

## 3. Results

### 3.1. The Correlation of HIV-1-RNA VL between Plasma and DPS Samples

**Figure 1:**
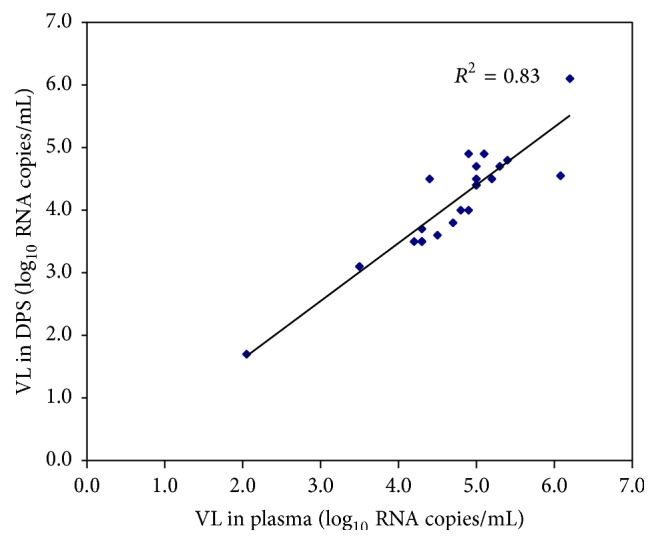
Log_10_ graph showing the linear regression comparing viral load data obtained from 22 matched samples of DPS versus plasma. log_10_ measurements of the HIV-1 RNA in DPS were assessed by NucliSens NASBA and in plasma samples were assessed by COBAS AMPLICOR HIV-1 Monitor. The values for the DPS are plotted against the values for the matched plasma samples. The linear correlation between the two samples is shown (*R* ^2^ = 0.83).

The mean VL was 4.7 log_10_ copies/mL (range 2.1 to 6.2) in liquid plasma samples and 4.1 log_10_ copies/mL (range 1.7 to 6.1) in DPS samples; thus, the difference in VL values was 0.6 log_10_ copies/mL, which was not statistically significant (*p* = 0.26). The data from 22 paired specimens are shown in Table 1. The linear regression analyses of the log-transformed RNA measurements (log_10_ copies/mL) for paired specimens of HIV-1 RNA VL in liquid plasma versus DPS samples (Figure 1) showed a good Pearson correlation (0.83). Only a single sample (HIV-03) produced a difference between the VL values of above 1.0 log_10_.

### 3.2. PCR Amplification of the HIV-1* pol* Sequence in DPS and Plasma Paired Samples

The average storage time for 21 out of the 22 samples that were assayed was 7.3 days, whereas one DPS sample (HIV-22) was stored for 910 days, just to see the amplification of a sample stored for a long period of time (Table 1). The 1.1 kb fragment of HIV-1* pol* was amplified in 63.6% (14/22) of the 22 paired samples. All samples that could be amplified from DPS were also amplified in their paired liquid plasma, with the exception of sample HIV-55. Only one sample (HIV-88) could be amplified from liquid plasma but not from DPS.

### 3.3. Comparison of DPS and Plasma-Derived Consensus* pol* Sequences Obtained from Patient Specimens Using an In-House Assay

**Figure 2:**
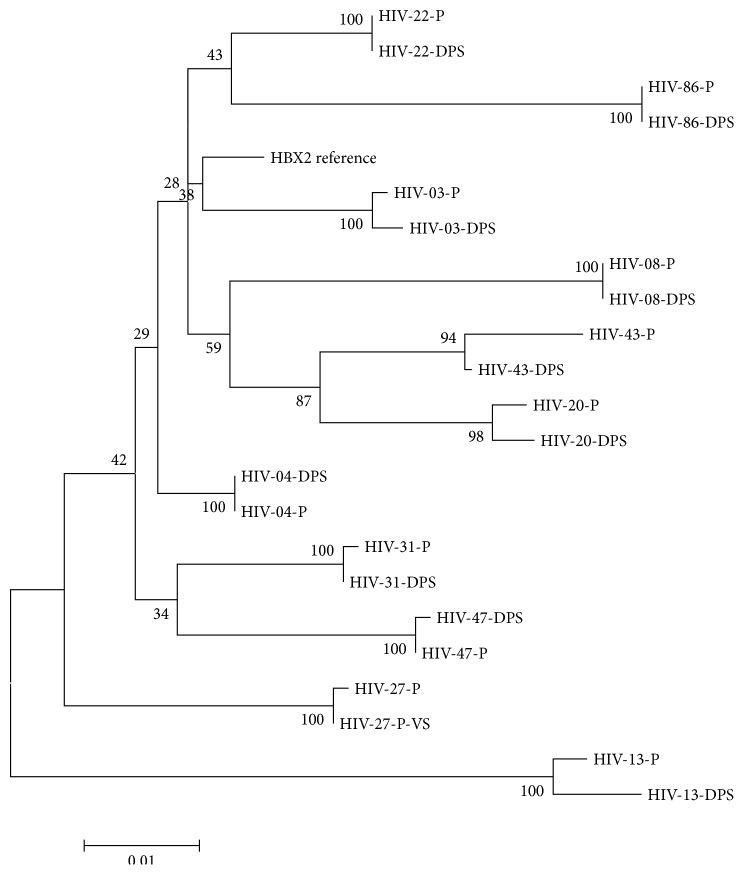
Phylogenetic tree constructed using sequence data generated from 22 HIV* pol* region paired plasma (P) and DPS (D) samples. The tree incorporating 1000 nonparametric bootstraps, constructed using sequence data from paired plasma and DPS samples. Phylogenetic tree was produced by the neighbor-joining method and the reliability of the branching orders was assessed by using the bootstrap approach with Mega 6.06 software [10]. One reference sequence representing HIV subtype B was included (HXV2-B subtype). Paired samples (P) or DPS are indicated in the patient ID. Sample HIV-27, which was previously genotyped with ViroSeq (VS), was included and paired with the sequence obtained with the in-house genotyping assay from the plasma sample.

To assess the reproducibility of the in-house assay compared to a commercial method (ViroSeq), the HIV RNA from sample HIV-27, which was previously genotyped with ViroSeq, was amplified with the in-house method and the resultant sequence was compared against the HIV-1* pol* sequence. The phylogenetic analysis showed a high correlation between the sequences (Figure 2).

A total of twenty-two HIV-1* pol* sequences were analyzed to determine the percentage of their similarity using the Mega 6.06 program. The mean similarity between HIV* pol* nucleotide sequences that were obtained from plasma and DPS samples was 98.3%, with a range of 95% to 99%. All sequenced samples corresponded to HIV-1 subtype B. A phylogenetic tree derived from the 10 paired HIV-1* pol* sequences showed a close clustering of sequences from the same paired samples in all cases (Figure 2). The bootstrap values that were measured for the paired plasma/DPS sequences were higher than 99% in almost all of the samples. A high similarity between deduced amino acid sequences was also noted, which indicated a high concordance between HIV-1* pol* sequences that were obtained from paired plasma and DPS samples.

### 3.4. Comparison of Drug Resistance Mutations in Paired Plasma versus DPS Samples

A total of ten paired DPS/plasma samples were analyzed, and the presence of DRM and susceptibility to ARV drugs were both determined using the Stanford HIV database. The mutations associated with DR and the genotype interpretation of 20 plasma and DPS-derived PR and RT gene sequences are shown in Table 2. A total of 60% (6/10) of the paired samples contained DRM in the PR and/or RT genes. There was no difference in the DRM found between plasma and DPS samples in seven of the paired samples. Three samples (HIV-13, HIV-20, and HIV-22) indicated some degree of discordance (Table 2). For instance, in one case, the L10F mutation in the PR gene was detected in the sample derived from plasma but was not detected in the DPS counterpart; thus, the interpretation of resistance did not change. In another case, the T215Y mutation in the RT gene was only present in the DPS sample but not in the plasma counterpart; again, there was no change in the interpretation of resistance.

## 4. Discussion

In recent years, there have been efforts to expand the monitoring of ARV treatment into LMICs, where more than 90% of new HIV infections are diagnosed; for example, regions in sub-Saharan Africa account for 70% of all AIDS-related deaths [12]. The high costs of the assays that are used to evaluate VL and drug-resistant genotypes present a hurdle to the successful monitoring of ARV therapy; these costs derive from the logistics that are involved in the transport, storage, and processing of blood samples in reference laboratories. In the majority of LMICs, there are few laboratories with the infrastructure and staff skills needed to analyze these samples and most of them are not located near the clinics where patients are following up; therefore, measurements of VL and/or the evaluation of DRM are not routinely performed. Consequently, the WHO criteria that are used to detect treatment failure are based on clinical and immunological parameters. However, studies have shown that these criteria lack the sufficient sensitivity and specificity that are needed to detect true virological failure, which increases the potential that HIV patients undergoing ARV therapy may accumulate numerous DRM, thereby reducing the effectiveness of therapeutic options [17, 18].

The use of DBS samples has been shown to be a safe, robust, and convenient method for the collection, transport, and storage of the small volumes that are used in the analysis of biomarkers [18]. DBS samples have also been used to measure VL [11, 19–26] and assess drug resistance genotypes [13, 14, 21, 27–29] in HIV-1 patients. The preparation of DBS samples does not require a centrifuge and can therefore be performed by unskilled personnel. However, the use of these samples also presents drawbacks, including the overestimation of VL levels due to the presence of proviral DNA within the blood mononuclear cells of the peripheral blood [14, 26], which is responsible for 33–80% of the amplification success obtained from these samples [14, 23, 30]. Another disadvantage of using DBS samples is the need for an appropriate method of removing PCR inhibitors from erythrocytes [31]. Unlike DBS samples, DPS samples provide the advantage of producing VL measurements that are comparable to that of liquid plasma (the gold standard of such measurements) because the contribution of proviral DNA is eliminated in this context. However, standard genotyping assays use plasma for this purpose, and it is notable that DRM appear earlier in plasma than in proviral DNA [32]. Thus, the sensitivity in detecting early treatment failure would be increased when assaying DPS versus DBS samples [5]. It is important to reach the lowest limit of VL detection when analyzing DBS samples. The WHO criteria indicate that in developing countries a VL of 3.7 log_10_ copies/mL is acceptable because the possibility of disease progression is low in patients with a VL below this value [33].

In this study, we were able to measure samples with a VL of >1.7 log_10_ copies/mL. We found a correlation between the VL of the liquid plasma samples (4.7 log_10_) and the DPS samples (4.1 log_10_); this 0.6 log_10_ difference was similar to that observed in other studies, in which differences have been measured within the range of 0.077 log_10_ copies/mL [34] to 0.64 log_10_ copies/mL [20]. Therefore, our results suggest that DPS samples can be used to monitor VL in HIV-1 patients. Although there was a >1 log_10_ difference in the VL of one sample in our collection, this was likely a result of either an error during sample preparation or the degradation of the RNA that was extracted from the DPS.

The stability of nucleic acids on filter paper becomes compromised over time and is also affected by the temperature and humidity at which the samples are stored. Previous studies have shown that samples that are stored for long periods of time are inadequately amplified [35]. However, there is also research that indicates that prolonged periods of storage, ranging from one year [29] to up to 5-6 years, did not significantly affect the success of genotyping. In our study, the DPS sample HIV-22 that was stored for 910 days (>2 years) was successfully amplified and sequenced for DRM [36]. Therefore, it seems feasible that these samples could be collected well in advance of being sent to a laboratory, which presents another advantage of using dried fluid spots as samples from patients living in rural areas. In central laboratories, these samples could even be archived for future studies. Additional research is needed to investigate how storage conditions affect DPS samples, particularly with respect to storage temperature and the length of storage time.

The effectiveness of amplification was 61.9% (13/21) in the group of samples that were stored for an average time of 7 days, demonstrating that DPS samples can be used for the RT-PCR amplification of HIV-RNA. Previous research has shown that the VL is a key determining factor of the effectiveness of amplification in DBS samples; using commercial genotyping methods, effective amplification was achieved in DPS samples with a VL above 15,000 copies/mL (4.1 log_10_) [37] and in DBS samples with VLs above 6,000 (3.7 log_10_) [38], 10,000 (4 log_10_) [29], and 14,000 copies/mL (4.1 log_10_) [15]. In our collection of samples that were stored for 7 days, it was possible to amplify the 1.1 kb fragment in the samples that had a VL above 4000 copies/mL (3.6 log_10_). It has been previously reported that amplification success is lower when using DPS versus DBS samples. For example, it has been reported that using an in-house RT-PCR assay to amplify a 1023 bp fragment ofHIV-1* pol* gene was possible in DPS samples with a VL of 338,112 copies/mL (5.5 log_10_) that were stored for 6 years at −30°C and in DPS samples with a VL of 57,375 copies/mL (4.7 log_10_) that were stored for 5 years at −70°C. In DBS samples, amplification was possible with even lower VL values: 6,452 copies/mL (3.8 log_10_) and 19,497 copies/mL (4.2 log_10_), respectively [36]. It has been reported that the success of RT-PCR amplification is greater when using an in-house assay compared to commercial methods such as ViroSeq (Abbott Molecular, IL, USA) and TruGene (Siemens Healthcare Diagnostics, IL, USA) [24]. The use of in-house RT-PCR assays is advantageous to developing countries because commercial testing costs approximately $230 and the rate of amplification is lower [24]. Inzaule et al. reported that the cost of commercial genotyping testing decreased from $278.31 to $110.05 for DBS samples, which represents a 60% reduction in cost [16].

In agreement with what was previously reported by McNulty et al. [36], in our study we could amplify a fragment of the HIV-1* pol* gene from 100 *μ*L of plasma (two DPS circles). We used samples that had been previously sequenced (HIV-22 and HIV-27) [15] to compare against the sequences that were obtained using the in-house assay, and both sets of sequences were found to be highly similar.

Overall, we found a good concordance between the genotyping of liquid plasma samples and the paired DPS samples; the variability that arose in the context of DR mutations was not relevant to the interpretation of the genotypic algorithm and was similar to that found in other studies using DBS samples [39, 40].

The patients included in this study had not been previously tested for HIV drug resistance. Although this study did not include follow-up with the patients, it is notable that a high percentage of DRM was found, which will likely impact the success of ARV therapy. This finding highlights the need to improve the monitoring of HIV patients who are undergoing ARV therapy in Mexico. Because a patient with DRM can transmit a resistant virus which can lead to unsuccessful responses to ARV therapy; the early detection of viral mutations would enable a second-line regimen of ARV drugs to be administered in a timely manner and might prevent the accumulation of DRM.

One limitation of the current study was our inability to identify what caused amplification failure in several samples with high VL, which suggests that the mishandling of these samples might have affected their amplification success. The proper handling, storage, and laboratory processing of DPS samples are critical components to the success of drug resistance genotyping.

Future studies comparing the different methodologies used during each step of sample analysis (RNA extraction, cDNA synthesis, amplification, primers, PCR, sequencing, etc.) may help to identify a strategy that can improve amplification efficiency, which could in turn improve the ability to accurately measure VL in samples with low VL (<15,000 copies/mL (4.1 log_10_)), as has been demonstrated by Monleau et al. (2010) [14]. The choice of RNA extraction method is critical to successful amplification with RT-PCR. In the case of RNA viruses, the use of a “one-step” RT-PCR assay is advantageous because it reduces the length of testing time; however, because the costs are higher with this method, it is not a feasible choice for the majority of laboratories that are located in LMICs. Until the infrastructure problems of our laboratories are resolved, using an in-house method may be the only way to improve the accessibility of drug resistance testing for HIV patients. Because the vast majority of these patients have access to ARV treatment, the early detection of drug-resistant virus is necessary to avoid patient losses.

In conclusion, the data obtained in our study demonstrate the feasibility of using DPS samples as a means of monitoring ARV treatment and disease progression. In an ideal scenario, peripheral laboratories could handle the centrifugation of blood samples and the preparation of DPS samples, which would then be mailed within seven days of their preparation to central laboratories where they could be stored at −20°C until further tested. The ability to efficiently monitor drug resistance in countries with the highest rates of HIV infection would provide a major public health tool for assessing the prevalence of HIV drug resistance in particular regions of the world and could help set guidelines for ARV treatment if necessary. Future research into standardizing the methodology used to process DPS samples could improve the monitoring of ARV treatment for patients living with HIV infection, especially if this method could be adopted for large-scale use.

## Figures and Tables

**Table 1:** Rate of HIV *pol* sequences PCR amplification from plasma and DPS paired samples.

ID	Time of DPS storage (days)	VL^*∗*^ (log_10_ copies/mL)		PCR amplification		DR genotype^*∗∗*^	
		Plasma	DPS	Plasma	DPS	Plasma	DPS
HIV-47	3	6.2	6.1	+	+	+	+
HIV-03	7	6.1	4.6	+	+	+	+
HIV-43	7	5.4	4.8	+	+	+	+
HIV-08	7	5.3	4.7	+	+	+	+
HIV-86	7	5.2	4.5	+	+	+	+
HIV-31	7	5.1	4.9	+	+	+	+
HIV-55	7	5.0	4.7	−	+	+	−
HIV-64	7	5.0	4.4	+	+	+	+
HIV-88	12	5.0	4.4	+	−	−	+
HIV-20	7	5.0	4.5	+	+	+	+
HIV-13	12	4.9	4.9	+	+	+	+
HIV-18	7	4.8	4.0	+	+	+	+
HIV-54	7	4.7	3.8	−	−	−	−
HIV-89	7	4.5	3.6	+	+	+	−
HIV-66	7	4.3	3.5	−	−	−	−
HIV-69	7	4.3	3.7	−	−	−	−
HIV-62	7	4.3	3.5	−	−	−	−
HIV-39	7	4.2	3.5	−	−	−	−
HIV-04	7	4.9	4.0	+	+	+	+
HIV-02	7	3.5	3.1	−	−	−	−
HIV-01	7	2.1	1.7	−	−	−	−
HIV-22^¹^	910	4.4	4.5	+	+	+	+

Average ± SD		4.8 ± 0.9	4.2 ± 0.9	14/22	14/22	14/22	13/22

Rate of PCR amplification and genotyping (%)				63.6			59.1

^*∗*^VL in DPS and liquid plasma using Amplicor HIV-1 Monitor Test, version 1.5.

^*∗∗*^Stanford genotyping drug resistance interpretation algorithm (v.4.2.6) (http://sierra2.stanford.edu/sierra/servlet/JSierra). DR: drug resistance genotype.

^¹^HIV-DPS sample stored for 910 days.

**Table 2:** Drug resistance mutations in the protease (PR) and reverse-transcriptase (RT) regions of HIV-1 identified in plasma and DPS paired samples.

Sample ID	HIV-1	Codons		Major drug resistance mutations
	Subtype	PR	RT	
HIV-03-P	B	21–99	1–259	RT (RTNI): K219Q
HIV-03-DPS		22–99	1–249	RT (RTNI): K219Q

HIV-04-P	B	21–99	1–259	None
HIV-04-DPS		3–99	1–259	None

HIV-47-P	B	9–99	1–251	None
HIV-47-DPS		15–99	1–248	None

HIV-43-P	B	6–99	1–254	RT (RTNNI): V179D
HIV-43-DPS		36–99	1–230	RT (RTNNI): V179D

HIV-08-P	B	4–99	1–254	RT (RTNNI): V108I
HIV-08-DPS		3–99	1–249	RT (RTNNI): V108I

HIV-86-P	B	3–99	1–254	None
HIV-86-DPS		15–99	1–249	None

HIV-31-P	B	3–99	1–259	None
HIV-31-DPS		42–99	1–239	None

HIV-20-P	B	22–99	1–250	PR (PI): V82A^*∗∗*^
HIV-20-DPS		16–99	1–239	PR (PI): V82A^*∗∗*^, I50M^1^

HIV-13-P	B	38–99	1–254	PR (PI): M46L^*∗∗*^, V82A^*∗∗*^, K43T^*∗*^, A71T^*∗*^, and G73C^*∗*^RT (RTNI): M41L, D67N, L74V, M184V, L210W, and K219Q RT (RTNNI): Y188L
HIV-13-DPS		4–99	1–259	PR (PI): M46L^*∗∗*^, I54V^1^, V82A^*∗∗*^, K43T^*∗*^, A71T^*∗*^, and G73C^*∗*^RT (RTNI): M41L, D67N, L74V, M184V, L210W, *T215Y* ^1^, and K219Q RT (RTNNI): Y188L

HIV-22-P	B	1–99	1–332	PR (PI): N88S, *L10F* ^2^, and M46V RT (RTNI): M41L, M184V, L210W, and T215Y
HIV-22-DPS		16–99	1–254	PR (PI): N88S, M46V RT (RTNI): M41L, M184V, L210W, and T215Y

RTNNI: nonnucleoside reverse-transcriptase inhibitors; RTNI: nucleoside reverse-transcriptase inhibitors; and PI: protease inhibitors.

^*∗∗*^Major drug resistance mutations to PI.

^*∗*^Minors drug resistance mutations to PI.

P = liquid plasma sample.

DPS = dried plasma spot sample.

^1^Not present in DPS paired sample; ^2^not present in plasma paired sample.
